# Avoidance of tracheal intubation using awake prone positioning in a case of severe respiratory failure from lung adenocarcinoma

**DOI:** 10.1097/MD.0000000000046989

**Published:** 2026-01-09

**Authors:** Rong Jiang, Haibo Zhou, Zhiguo Zhou

**Affiliations:** a Department of Respiratory and Critical Care Medicine, The First Hospital of Changsha (The Affiliated Changsha Hospital of Xiangya School of Medicine, Central South University), Changsha, Hunan Province, China; b Department of Respiratory and Critical Care Medicine, University of South China Hengyang Medical School, Hengyang, Hunan Province, China.

**Keywords:** awake prone position, case report, lung adenocarcinoma, lung cancer, tumor immune microenvironment

## Abstract

**Rationale:**

The awake prone position (APP) has been recognized for its efficacy in decreasing mortality rates among patients suffering from acute respiratory distress syndrome (ARDS). While APP is frequently employed in non-intubated ARDS patients, its utilization in individuals with lung adenocarcinoma to boost oxygenation is not widely documented.

**Patient concerns and Diagnoses:**

A 61-year-old male presented with a 2-month history of cough, sputum, dyspnea, and weight loss. Upon examination, his oxygen saturation was recorded at 80%, and auscultation indicated the presence of numerous moist rales in both pulmonary fields. Chest computed tomography (CT) revealed extensive ground-glass opacities, thickened septa, and right lower lobe consolidation. Bronchoscopy showed a substantial amount of clear, frothy sputum in both lungs; however, polymerase chain reaction analysis of the bronchoalveolar lavage fluid did not identify any microorganisms. A transbronchial lung biopsy confirmed the diagnosis of lung adenocarcinoma.

**Interventions:**

Given the patient’s deteriorating respiratory condition, it was determined to initiate prone ventilation. Remarkably, after 30 minutes of commencing prone ventilation, there was a significant improvement in his oxygenation levels. A subsequent reassessment of the lung CT after 2 days of treatment in the prone position demonstrated a reduction in lung lesions and an enhancement in ventilation compared to the initial presentation.

**Outcomes:**

After 1 week of daily APP (12 hours/d), oxygenation improved sufficiently to avoid intubation. Following genetic testing results, targeted therapy with vemurafenib was started. The patient was discharged after 1 week of combined treatment. A 1-month follow-up CT demonstrated substantial resolution of pulmonary lesions.

**Lessons:**

This case report details the management of a patient with lung adenocarcinoma complicated by acute respiratory failure, who underwent innovative awake prone positioning ventilation. After daily APP treatment for 12 hours over 1 week, significant improvement in oxygenation was observed, and tracheal intubation was successfully avoided. Following 1 week of combined treatment with the targeted drug vemurafenib, the patient was discharged without complications. A follow-up CT scan 1 month later revealed substantial resolution of the pulmonary lesions. Early use of the APP may be a potential treatment option for patients with respiratory failure from lung adenocarcinoma that may avoid progression to tracheal intubation.

## 1. Introduction

Prone positioning has become a standard evidence-informed practice for patients on mechanical ventilation who are experiencing moderate-to-severe acute respiratory distress syndrome (ARDS),^[1]^ but its application as an innovative intervention for spontaneously breathing patients, particularly those with lung cancer who do not have acute respiratory failure, is still relatively uncharted territory. From a physiological perspective, prone positioning could facilitate the recruitment of lung tissue in the posterior dorsal areas, primarily by reversing atelectasis.^[2]^ Prone positioning can lead to a more uniform distribution of ventilation, which may decrease lung strain due to changes in pleural pressure and the distribution of pleural space.^[3]^ This approach can enhance the ventilation-perfusion ratio^[4]^ and reduce shunting, which is the diversion of blood away from ventilated areas of the lung. We present a case of a patient with lung adenocarcinoma who was on the verge of requiring tracheal intubation due to non-acute respiratory failure. The patient experienced a temporary improvement in oxygenation with the combination of high-flow nasal oxygen therapy and awake prone positioning, creating an opportunity for subsequent targeted tumor therapy. Eventually, the patient’s condition improved significantly after targeted therapy, leading to their discharge. We present a detailed case of a patient with lung adenocarcinoma with respiratory failure where the systematic application of awake prone position (APP) was associated with a successful avoidance of intubation, and we discuss the potential role of this strategy in the oncologic population.

## 2. Case presentation

A 61-year-old male with xanthoderma, previously in good health, arrived at our hospital with a 2-month history of a cough, sputum, dyspnea, and weight loss. Notably, he did not have fever or hemoptysis. Initially, he was treated at the Xiangdong Hospital Affiliated to Hunan Normal University in Hunan Province. A lung computed tomography (CT) scan revealed bilateral infectious foci, while fiberoptic bronchoscopy showed clear airways without any constriction, bleeding, and a small amount of white, thin sputum in both lungs. Polymerase chain reaction testing of the bronchoalveolar lavage fluid found no bacteria, fungi, or tuberculosis, leading to a diagnosis of “pulmonary infection.” The patient was given a 4-day course of “cefoperazone-sulbactam sodium (3g, intravenous infusion, every 8 hours) + levofloxacin injection (0.5, intravenous infusion, daily)” antibiotic treatment. The patient’s symptoms of cough, expectoration, and dyspnea showed a slight improvement. However, after being discharged at his own request, the patient experienced a steady deterioration in dyspnea, accompanied by a cough that produced a small amount of white sputum. The patient sought treatment at a local clinic with traditional Chinese medicine, yet there was no significant improvement in his symptoms of cough and dyspnea. Five days ago, the patient’s dyspnea has become markedly worse, with mild exertion leading to increased respiratory distress. The patient’s daily activities had become unmanageable, and for the past 2 months, the patient had not experienced fever, chest pain, or hemoptysis, and had lost approximately 2 kg in body weight. Consequently, the patient came to our hospital seeking further treatment.

Upon assessment, the patient’s vital signs were noted as follows: body temperature of 36.7°C, a respiratory rate of 27 breaths per minute, blood pressure at 130/60 mm Hg, heart rate of 102 beats per minute, and peripheral oxygen saturation of 90%. The patient was alert, and auscultation of the lungs revealed numerous moist rales, with no appreciable pleural friction rubs. The cardiac and abdominal examinations yielded no abnormalities.

**Figure 1.**
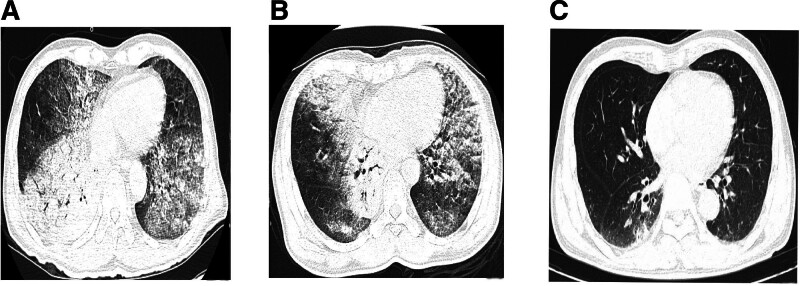
Chest CT: (A) On admission in the supine position showed diffuse ground-glass opacities in both lungs, thickened interlobular septa, and consolidation in the right lower lobe; (B) in the prone position after 2 days of APP; (C) in supine position 1 month after targeted therapy. APP = awake prone position, CT = computed tomography.

Upon admission, a blood culture was obtained, and the results were as follows: the white blood cell count was elevated at 10.41 × 10^9^/L (reference range: 4–10 × 10^9^/L), with a neutrophil percentage of 87.3% (reference range: 40%–75%). Additional laboratory tests, including alpha-fetoprotein, carcinoembryonic antigen, brain natriuretic peptide, C-reactive protein, and procalcitonin levels, all fell within the normal ranges. Arterial blood gas analysis revealed a partial pressure of oxygen of 57.7 mm Hg, a partial pressure of carbon dioxide of 32.3 mm Hg, and an oxygenation index of 128 mm Hg. Chest CT scans in the supine position demonstrated extensive ground-glass opacities throughout both lungs, with thickened interlobular septa and consolidation in the right lower lobe (Fig. 1A).

**Figure 2.**
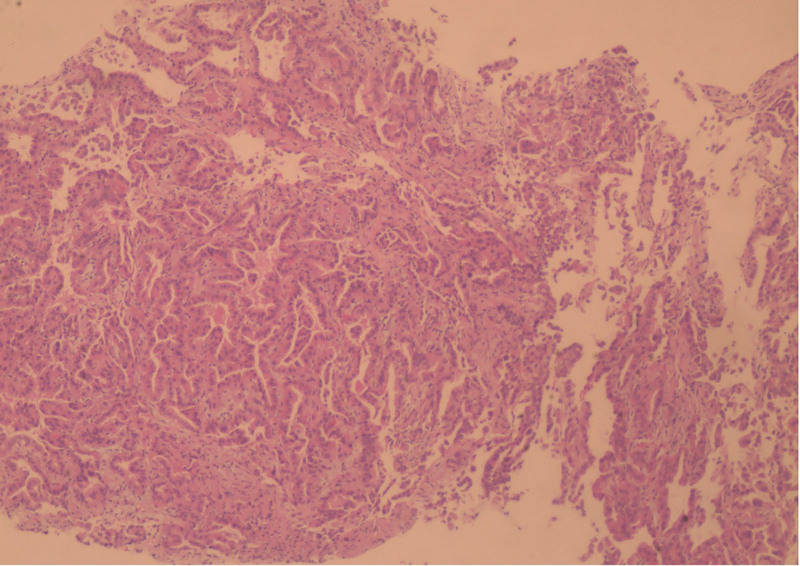
(Transbronchial lung biopsy [TBLB], right lower lobe) adenocarcinoma, mostly adherent growth, local infiltration.

Given the absence of fever and the normal levels of inflammatory markers such as procalcitonin and C-reactive protein, we considered the possibility of a noninfectious condition like “organizing pneumonia” and did not rule out lung cancer. This patient has not been diagnosed with lung cancer and has not received treatment with immune checkpoint inhibitors. From the medical history, immune checkpoint inhibitor-related pneumonitis can be excluded. The patient was temporarily administered high-flow nasal cannula (HFNC, fraction of inspired oxygen 45%, flow 45L/min), along with levofloxacin injection (0.5, intravenous, once daily) and methylprednisolone sodium succinate injection (40mg, intravenous, once daily) for a period of 4 days. The patient’s persistent shortness of breath, with no relief in sight, left the diagnosis of the lung condition shrouded in uncertainty. Following the approval from the patient’s family, we opted to conduct a bronchoscopy, acknowledging the inherent risks. The procedure exposed a substantial accumulation of clear, frothy sputum in both lungs; nevertheless, the polymerase chain reaction testing of the bronchoalveolar lavage fluid yielded no detection of microorganisms. Despite the patient exhibiting atypical clinical symptoms, the definitive pathological diagnosis confirmed the presence of lung adenocarcinoma (Fig. 2). We ceased hormone therapy and proceeded with symptomatic treatment using levofloxacin for anti-infection, as well as antitussive and expectorant therapies. The patient’s diagnosis of lung adenocarcinoma was confirmed. But the results of genetic tests will not be available for about a week. The patient’s family declined chemotherapy and the use of targeted drugs without genetic guidance. In this scenario, the patient’s condition worsened, as evidenced by a deteriorating oxygenation status indicated by a oxygenation index (OI) below 100 mm Hg. In response, the fraction of inspired oxygen (FiO_2_) was increased to 90% under HFNC with a flow rate of 45 L/min, and heated and humidified gas (temperature 36°C, humidity 100%) was used. The patient still felt short of breath and had mild pain in the nasal mucosa, which was tolerable. Despite these efforts, maintaining normal oxygen saturation levels remained a challenge. The patient exhibited a rapid respiratory rate of 35 to 40 breaths per minute, signaling that tracheal intubation was imminently necessary. Given the patient’s escalating respiratory distress, the decision was made to implement prone ventilation. When initiating prone positioning ventilation, the attending physician provides bedside guidance. The patient lies face down on the bed with their body aligned parallel to the bed surface. The head of the bed is elevated by 30°C to prevent gastric reflux. A soft pillow supports the forehead to avoid pressure on the eye sockets and zygomatic bones. A 5 to 10 cm thick cushion under the sternum creates thoracic cavity elevation. A soft pillow at the anterior superior iliac spine elevates the abdomen. Both upper limbs are flexed at over 90 degrees at the elbows, positioned laterally to the head. Knees are bent at 15 degrees with ankles naturally hanging down. The patient is instructed to support their forehead with both hands on pillows to enhance tolerance. After 30 minutes of starting this intervention, there was a marked enhancement in the patient’s oxygenation levels. The patient’s arterial blood gas levels showed significant improvement after a 2-hour interval, with a pH level of 7.35, carbon dioxide at 36 mm Hg, oxygen partial pressure at 70 mm Hg, oxygen saturation at 95%, and a OI exceeding 85 mm Hg. The next day, the patient continued with complete prone positioning ventilation while maintaining HFNC (FiO_2_ 80%, Flow 40 L/min) to sustain oxygen saturation. The patient demonstrated excellent compliance, completing 6 hours of prone ventilation under medical supervision. Following each 2-hour session, the patient was repositioned supine for a 30-minute rest period before APP. To enhance adherence, the prone ventilation protocol was conducted during daytime hours. The patient showed good tolerance with no adverse reactions reported throughout the treatment course. A chest CT scan in the prone position after 2 days of APP is depicted in Figure 1B. Compared to the supine position, the lung lesions in the prone position showed a tendency to decrease. On days 3 to 6, the patient increased the time of prone ventilation to a total of about 12 hours per day. The duration of complete prone ventilation was determined according to the patient’s own comfort and compliance, but the total time per day was guaranteed to be more than 12 hours. During the whole process, the patient did not have any APP related adverse reactions, such as pressure injury, circulatory fluctuation, reflux aspiration and other problems. With the application of approximately 12 hours per day of prone positioning treatment, the fraction of inspired oxygen was gradually reduced to 50%, accompanied by a steady improvement in oxygenation levels. After 7 days of high-flow oxygen inhalation and levofloxacin for anti-infective treatment in the prone position, the patient’s genetic test results were returned. The tests revealed a significant EGFR mutation abundance of 73.23%. Additionally, the programmed cell death 1-ligand 1 (PD-L1) immunohistochemical test results indicated a Tumor Proportion Score of <1% and a Combined Positive Score of <1 Following discussions with the patient’s family, they opted for the targeted therapy of Furmonertinib Medilate Tablets (2 pills once daily), a cost-effective choice. Within 2 days of commencing the medication, there was a notable improvement in the patient’s cough and shortness of breath, and the OI further rose to 200mmHg. Considering that the effects of epidermal growth factor receptor-tyrosine kinase inhibitors (EGFR-TKI) therapy are usually observed over weeks, this early improvement is more likely attributable to APP and high-flow oxygen support. The patient’s oxygen delivery was transitioned to low-flow inhalation, and after 1 week of targeted therapy, the patient was discharged. Postdischarge, the patient continued on Furmonertinib Medilate Tablets (2 pills once daily) for 1 month, and a significant absorption of the lung lesions was observed upon reevaluation of the lung CT (Fig. 1C). The patient feels well with no fever, cough, sputum production, hemoptysis, or obvious dyspnea. He is able to perform light physical activities and can fully take care of their daily life. Table 1 summarizes the diagnosis and treatment process of this case after admission to our hospital, as well as APP and HFNC parameters and changes in oxygenation.

**Table 1:** Timeline of diagnosis and treatment process and oxygenation changes.

Time	Event	HFNC (FiO_2_)	PaO_2_ (mm Hg)	PaO_2_/FiO_2_ (mm Hg)	APP	Diagnostic and therapeutic measures
Days 1–4	Admission and initial treatment	45%	57.7	128	Not implemented	Levofloxacin injection (0.5 ivgtt, Qd), methylprednisolone sodium succinate injection (40mg, ivgtt, Qd)
Day 5	Bronchoscopy, pathological diagnosis of lung adenocarcinoma	50%	80	160	Not implemented	Levofloxacin injection (0.5, ivgtt, Qd)
Day 6	The condition has worsened further	90%	60	66	Not implemented	Levofloxacin injection (0.5, ivgtt, Qd)
Day 7	Start APP	80%	70 (After 2 hours of APP)	87 (After 2 hours of APP)	2 h	Levofloxacin injection (0.5, ivgtt, Qd)
Days 8–14	Continue APP	50%	80	160	6–12 h/d	Levofloxacin injection (0.5, ivgtt, Qd)
Days 14–15	Know the genetic testing results	50%	90	180	12 h/d	Furmonertinib Medilate Tablets (2 pills once daily)
Days 16–20	Targeted therapy	40%	80	200	12 h/d	Furmonertinib Medilate Tablets (2 pills once daily)
Day 21	Discharge	30%	85	283	Unused	Furmonertinib Medilate Tablets (2 pills once daily)
One month after discharge	Follow-up examination	Unused	Normal	Normal	Unused	Furmonertinib Medilate Tablets (2 pills once daily)

APP = awake prone position, FiO_2_ = the fraction of inspired oxygen, HFNC = high-flow nasal cannula, ivgtt = intravenous guttae, PaO2 = partial pressure of oxygen, PaO₂/FiO₂ = oxygenation index, Qd = quaque die.

## 3. Discussion

The combination of APP with noninvasive ventilation or high-flow oxygen therapy can serve as an effective and secure method for treating hypoxemic acute respiratory failure.^[5,6]^ Although the potential of APP to enhance oxygenation has been recognized, its application has not been extensively adopted in clinical practice until the outbreak of Severe Acute Respiratory Syndrome Coronavirus 2. A systematic review and meta-analysis synthesized data from 12 observational studies across the globe, encompassing 1156 non-intubated patients with ARF stemming from ARDS or corona virus disease 2019 (COVID-19) respiratory failure. The principal meta-analysis revealed a significant reduction in mortality rates and an improvement in the OI.^[7]^

Currently, lung cancer has a high incidence rate, and many patients develop severe respiratory failure without a prior diagnosis and face the need for tracheal intubation and mechanical ventilation to maintain oxygenation. In this case, the patient relied on traditional Chinese medicine for approximately 2 months, leading to a delay in appropriate treatment, which may have contributed to clinical deterioration. The surge in patients necessitating critical care has indeed placed substantial strain on intensive care unit resources, compelling clinicians to carefully balance available resources with the sudden demand. Initially, APP was adopted as a practical method to enhance oxygenation and potentially decrease the rate of tracheal intubation. However, the application of APP in non-COVID-19 patients with acute renal failure is still being explored.^[8,9]^

The patient we reported on had no known underlying health conditions and was primarily admitted with progressive shortness of breath. The symptoms of cough and expectoration were not prominent, and there was no indication of excessive sputum production. After receiving anti-infective treatment at another facility, there was a brief improvement in respiratory distress, leading to the patient’s discharge. However, no further treatment was sought, and the condition deteriorated postdischarge. The patient and family neglected the worsening symptoms and opted for traditional Chinese medicine for 2 months, which unfortunately delayed proper medical intervention. Upon arrival at our facility, the patient was in severe respiratory distress and was directly admitted to the respiratory intensive care unit. Radiological assessments indicated findings consistent with organizing pneumonia, and there was a transient improvement in dyspnea following steroid therapy, which initially led us to a misdiagnosis. The most critical diagnostic measure in this case was the temporary improvement in oxygenation after steroid treatment, so we took the risk of performing a fiberoptic bronchoscopy with high-flow oxygen. The bronchoscopy revealed a large amount of foamy sputum, which gave us a clue that the patient might have lung cancer. The pathological examination of the bronchial biopsy and the lung lavage fluid showed that the patient had adenocarcinoma. Initially, we leaned towards a diagnosis of lung adenocystic carcinoma, however, the definitive pathological findings indicated adenocarcinoma. The excessive mucus formation remains an enigma that has yet to be fully comprehended. This case underscores the significance of fiberoptic bronchoscopy and pathological examination of lung lavage fluid in unraveling the mysteries of lung diseases. For patients presenting with lung lesions of unknown origin, it is advisable to expedite the arrangement of bronchoscopy by experienced practitioners.

The likely cause of the patient’s poor oxygenation was the excessive mucus production by the lung cancer cells, which impaired the patient’s lung ventilation. Following the combination therapy of high-flow nasal oxygen and awake prone position (APP), the patient’s oxygenation improved temporarily. The potential reasons for this improvement are as follows:

In the prone position, the influence of gravity is leveraged to redistribute local pressures and gradients, which can help to re-expand areas of the lung that were previously non-aerated or poorly aerated. While the ventral lung, now in a dependent position, may undergo some degree of collapse or consolidation, this is typically less severe than the dorsal lung field. The positioning does not significantly alter regional lung perfusion, leading to better ventilation-perfusion matching and improved oxygenation.^[2]^ Consequently, prone positioning facilitates lung recruitment in the posterior, dependent lung regions by reversing atelectasis. In spontaneously breathing patients, this positioning technique encourages more uniform ventilation, attributed to reduced lung stretch and strain. The alteration in pleural pressure and the distribution of pleural space across different lung regions contribute to this effect.^[3,10]^ The enhancement in ventilation-perfusion matching is likely due to an improved distribution of physical forces, such as alterations in pleural pressure gradients and gravitational forces, as well as the distribution of transpulmonary pressure. Additionally, prone positioning mitigates the “pendelluft phenomenon,” which is the shift of gas from better-aerated, nondependent lung regions to less well-aerated, dependent regions.^[4]^ Uniformity in lung aeration, coupled with a stable perfusion pattern, can lead to a reduction in shunting. This mechanism underlies the potential benefits of short-term interventions, ultimately enhancing patient-centered outcomes like mortality rates. Although this study primarily focuses on patients with ARDS, its findings may provide some reference for understanding the potential benefits of prone positioning in lung cancer patients.

Drawing from the pathophysiological shifts observed with prone position ventilation, we infer that APP ventilation may not be universally applicable to lung cancer patients experiencing respiratory failure. When the primary radiologic feature is a lung mass or localized airway obstruction, prone position ventilation might not yield substantial benefits. On the other hand, in cases where lung cancer manifests as extensive ground-glass opacities, prone position ventilation could temporarily enhance oxygenation.

Awake proning is generally considered safe and has an added benefit of allowing patient-family interactions during hospitalization, which enhances the humanization of care.^[11]^ Ensuring patient compliance and tolerability is essential. While awake prone positioning has not been significantly associated with adverse events, it does have its constraints, such as the potential for intolerance, discomfort, and anxiety. Other limiting factors include obesity (particularly central obesity), pregnancy, and the crucial need for dedicated healthcare providers to assist in preventing patient injuries. Managing a conscious patient who is experiencing rapid deterioration can pose significant challenges at the ward level. Consequently, the success of awake proning, particularly during the initial sessions, relies heavily on the close collaboration and input from a multidisciplinary team comprising doctors, nurses, and physiotherapists. Patients must be well-informed about the procedure and willing to cooperate. Clinicians should consider practical aspects such as ensuring optimal pain management, maintaining clear communication, and providing assistance to enhance comfort, compliance, and accurate positioning.^[7]^ Nevertheless, we observed scant uniformity in the application of awake prone positioning, with a dearth of clarity regarding the most opportune time for initiation and the most effective duration of treatment.^[12,13]^ Additional studies are required to determine the optimal timing for initiating awake prone positioning and to clarify the specific stage at which it should be implemented, whether during mild or moderate hypoxia. Our patient opted for 12 hours of prone positioning daily, aligning with the Chinese guidelines for the diagnosis and treatment of novel coronavirus infection (10th edition).

This case study is distinctive as it represents the pioneering use of prone positioning in non-intubated individuals experiencing hypoxemic respiratory distress due to lung adenocarcinoma. Initially, the attention given to APP was driven by the belief that it could be a secure and efficacious method for managing ventilator scarcity amidst the peak of the Severe Acute Respiratory Syndrome Coronavirus 2 outbreak. Currently, there is a scarcity of documented cases regarding the use of the APP in type I respiratory failure attributed to lung adenocarcinoma. Our case indicates that APP serves as a beneficial supplementary treatment for respiratory failure in non-intubated patients suffering from lung adenocarcinoma, with early application proving to be beneficial. The efficacy of our therapeutic approach was attributed to the synergistic effect of APP and high-flow nasal oxygen therapy, coupled with the patient’s high level of compliance. This synergy provided the patient with the opportunity to proceed with tumor treatment. Ultimately, the patient’s respiratory failure was resolved, oxygenation was restored, and there was a significant reduction in lung lesions.

At the same time, whether the significant therapeutic effect obtained by the patient in the later anti-tumor treatment after prone position ventilation is related to prone position ventilation is also a direction for our consideration. In recent years, multiple clinical studies have found that prone position ventilation may affect the systemic immune response through mechanical conduction pathways. For example, in patients with ARDS, prone position ventilation can reduce the levels of plasma Interleukin-6 and Interleukin-8 and increase the expression of human leukocyte antigen (locus) DR in peripheral blood mononuclear cells, indicating its anti-inflammatory and immune regulatory effects.^[14]^ Mechanistically, the change in intrathoracic pressure gradient during prone position may affect lymphatic return and promote the migration of immune cells to the tumor site.^[15]^ In addition, animal experiments have shown that periodic mechanical stress can enhance the cytotoxicity of CD8 + T cells by activating the Piezo1 ion channel.^[16]^ These findings provide a theoretical basis for the application of prone position ventilation in tumor immunotherapy. Although the mechanism of mechanotransduction-immune interaction is gradually being revealed, no clinical studies have yet explored the effect of postural interventions (such as prone position ventilation) on the tumor microenvironment (TME) of lung adenocarcinoma. Current studies on PPV mostly focus on the improvement of respiratory mechanics and neglect its potential regulatory effects on the immune system.^[17]^ Whether prone position ventilation can reshape the tumor microenvironment of lung adenocarcinoma through mechanical signals needs to be further confirmed by large-scale clinical studies.

However, this case report as a single-patient case has inherent limitations. The therapeutic experience of a single patient may not be generalizable to broader populations due to individualized responses to treatment, and its conclusions necessitate further validation through large-scale studies. There is diagnostic uncertainty; although the patient’s initial presentation of rapidly progressing respiratory failure and imaging exudative shadows aligns with severe pneumonia characteristics, etiological differentiation (such as atypical pathogens or cryptogenic organizing pneumonia) is limited by detection sensitivity. Clinical improvement during treatment may mask underlying undiagnosed comorbidities, affecting the accuracy of attribution. Additionally, the absence of a control group introduces interference factors. The patient’s clinical improvement (e.g., enhanced oxygenation) could stem from the anti-inflammatory effects of glucocorticoids, while spontaneous disease resolution or synergistic drug interactions cannot be ruled out. The lack of a control group prevents distinguishing between treatment benefits and spontaneous remission, introducing confounding biases in efficacy evaluation. This case involves a young patient with high compliance, whose proactive behavior may amplify the treatment response, leading to an optimistic overestimation of the intervention’s effectiveness. Such selective characteristics are unlikely to be replicated in the general patient population, compromising the clinical applicability of the conclusions.

APP could be a promising and advantageous intervention, yet its significance may not be fully realized unless it is administered at the most opportune moment, for an appropriate duration, and to a suitable patient demographic. This approach is an economical and feasible method for managing patients in respiratory decline; however, it should be executed by skilled healthcare providers, and intubation should not be postponed if medically necessary. In summary, the application of APP in lung cancer patients is still in the research phase, and more clinical trials and studies are needed to determine the best application methods and potential benefits in this patient population. As research progresses, there may be more guidelines and recommendations to guide clinical practice in the future.

## Author contributions

**Conceptualization:** Zhiguo Zhou.

**Data curation:** Haibo Zhou.

**Writing – original draft:** Rong Jiang.

**Writing – review & editing:** Rong Jiang, Zhiguo Zhou.
